# Supplementary material for: Identification of photoperiod-induced specific miRNAs in the adrenal glands of Sunite sheep (Ovis aries)
Source: Front Vet Sci. 2022 Jul 22;9:888207. doi: 10.3389/fvets.2022.888207 (PMC9354845; doi:10.3389/fvets.2022.888207)
Supplement: Supplementary file 1 [file Data_Sheet_1.ZIP › Supplementary file/miRDeep2 file.zip!/miRDeep2 file/Novel_Structure/SPLP42A1/Novel_357.pdf]

Provisional ID	:	NC_019458.2_1085
Score total	:	2.3
Score for star read(s)	:	-1.3
Score for read counts	:	0
Score for mfe	:	2
Score for randfold	:	1.6
Score for cons. seed	:	
Total read count	:	96
Mature read count	:	94
Loop read count	:	0
Star read count	:	2

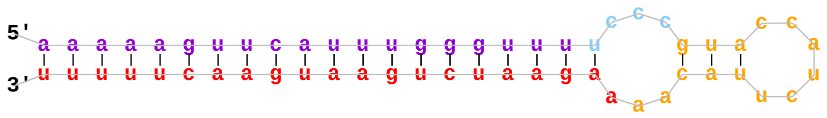

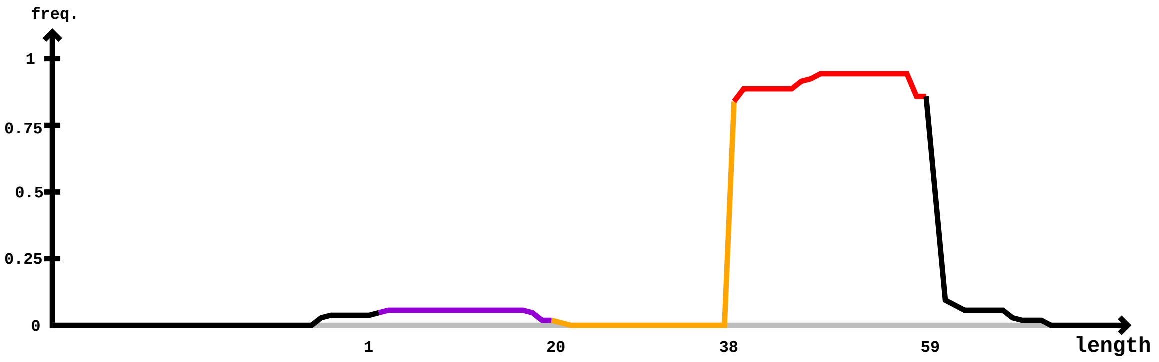

Star Mature

```
5'- caaaaugaaugaacuaugauauugcguuggccaaaaaguucauuuggguuuucccguaccaucuuacaaaagaaucugaaugaacuuuuuggccaacccaauacauucau   -3'    obs
    caaaaugaaugaacuaugauauugcguuggccaaaaaguucauuuggguuuucccguaccaucuuacaaaagaaucugaaugaacuuuuuggccaacccaauacauucau          exp
    ....(((((((........(((((.(((((((((((((((((((((((((((...(((......)))...))))))))))))))))))))))))))).))))))))))))   reads      mm        sample
    ..........................uuggGcaaaaaguucauuuggg..............................................................   1          1         seq
    ..........................uuggccaaaaaguucauuCgggu.............................................................   1          1         seq
    ..........................uuggccaaaaaguucauuugggG.............................................................   1          1         seq
    ...........................uggccaaaaaguucauuugAgu.............................................................   1          1         seq
    ................................aaaaaguucauuuggguuu...........................................................   1          0         seq
    .................................Uaaaguucauuuggguuu...........................................................   1          1         seq
    .....................................................................aaAaaucugaaugaacuuu......................   9          1         seq
    .....................................................................aaAaaucugaaugaacuuuuu....................   80         1         seq
    ......................................................................agaaucugaaugaacuuuuu....................   1          0         seq
    ......................................................................agaaucugaaugaacuuuuugg..................   2          0         seq
    ......................................................................aAaaucugaaugaacuuuuuggc.................   2          1         seq
    ............................................................................ugaaugaacuuuuuggccaacA............   1          1         seq
    ............................................................................ugaaugaacuuuuuggccaacc............   1          0         seq
    ............................................................................ugaaugaacuuuuuggUcaaccc...........   1          1         seq
    .............................................................................gaaugaacuuuuuggccaacccaUu........   1          1         seq
    ..............................................................................aaugaacuuuuuggccaacc............   1          0         seq
    ..............................................................................aaugaacuuuuuggccaacccaaG........   1          1         seq
```
